# Supplementary material for: Prevalence and socio-demographic correlates of accelerometer measured physical activity levels of school-going children in Kampala city, Uganda
Source: PLoS One. 2020 Jul 9;15(7):e0235211. doi: 10.1371/journal.pone.0235211 (PMC7347200; doi:10.1371/journal.pone.0235211)
Supplement: Attachment — Submitted filename: review comments.docx [file pone.0235211.s002.docx]

**Prevalence and sociodemographic correlates of accelerometer measured physical activity levels of school-going children in Kampala city, Uganda**

**Reviewer comments**

**Overall comment:** In this study, the authors describe the prevalence and examine the sociodemographic correlates of accelerometer measured physical activity levels among school-going children in Kampala, Uganda. Using a multistage random sampling method, the authors recruited 256 participants from 7 primary schools. In my view, this is an important study which is timely and provides much needed objective data on childhood physical activity in a LMIC. The school environment plays an important if not a very significant role in children’s physical activity behaviors. I have identified important issues that the authors should consider making to improve their manuscript. Some parts of the methods should be expanded and made clear for the readers. Please consider having another co-author read through your manuscript for editorial reasons. There are several small but meaningful errors such as acronyms and wrong brackets for references that should be addressed

**Introduction**

1. Once you have defined Physical activity as PA, Moderate-to-Vigorous Intensity Physical activity as MVPA etc., after the first time you use the acronyms, try to be consistent and do not revert to spelling out the whole word and vice versa. It is something the authors should address throughout the manuscript.
2. Please add “for children” on line 59: after the phrase…revised their PA guidelines.
3. Line 62: after health benefits, could you please add something like “associated with regular participation in PA.
4. Line 64: delete the word ‘also’ after Sallis and colleagues.
5. Add a comma after the phrase …literature shows that in children,…
6. References 13,14,15 on line 70, have different brackets to the rest of the references
7. The sentence starting on line 82 to 85 may need to be revised. Are you suggesting that all children have limited cognition, or do you mean limited reading comprehension skills due to their age?
8. When referring to children, it reads a bit odd to repeatedly say ‘in’ maybe consider using ‘among’
9. References 13,26,31, line 100 and reference 35 on line 103 have different form of brackets
10. Is HSES the same as SES? If so, please use the same acronym throughout your manuscript. Otherwise define HSES the first time you use it and then be consistent after that.
11. You use the acronym LICs on line 112, but I am not sure it has been defined before this mention
12. Line 115, consider rewording to say: Therefore, the present study….

**Methods**

1. Consider starting the methods section with “This is a cross-sectional study of a representative sample of school-going children aged 10-12 years old in Kampala, Uganda”.
2. Could you please substitute the word ‘tribes’ with perhaps ‘ethnic groups’? The word tribes has colonial connotations
3. How did you differentiate between awake non wear time and sleep time given that you are using a 24 hour protocol? Did you just lump possible sleep time and awake non wear time together because in your study you were not interested in measuring sleep duration? If so, please make that clear in your methods
4. There is debate about the accuracy of classifying children over 5 years old as being ‘underweight’ rather than being ‘thin’ based on BMI z scores. Have authors thought about the implications of this give their reference to WHO 2007 BMI percentiles which uses thinness for the age group being studied here?
5. Was the questionnaire validated for this population? If so please make that clear in your description of the questionnaire.
6. Please clarify your recruitment and response rate. Are you calculating the percentages of children with consent out of 400 or the 600? If you are calculating it from 600 then your percentages are off. The first one 66.7% is accurate but the next two need to be described accurately. For example you could say “of the 600 who were invited to participate, 400 (66.7%) had parents who completed questionnaires…etc.
7. Was there a specific reason for authors to use bivariate logistic regression as opposed to some hierarchical model which would be more robust to account for the clustering at the school and classroom levels?

**Results**

1. Generally, it may be better to avoid the use or terms such as ‘almost’ and rather use approximately, about, close to…etc
2. Be consistent, either use numbers or words when writing numerical data in text. For example ‘One in every 5 parents/guardians’…may be better to just say one in every five…
3. Do you mean approximately ‘equal numbers by age’? You have three ages and as such could not be ‘even’.
4. Table 1: Overweight is one word.
5. Consider using the ‘merge cells’ function to make your sub-heading lines one line in tables.
6. You are using children/participants interchangeably in your results section. Could you please pick one and be consistent with it.
7. Line 230: ‘Overall on average the children spent..’ seem awkward.. can you consider revising for readability?
8. The sentence on lines 251 starting with ‘Males…’ is awkward, consider rephrasing it.

**Discussion**

1. Do you think that it was overweight/obesity that caused the participants to be more sedentary or that it was the sedentariness that led to being overweight/obese? Reverse causality is a possibility isn’t it? What is your comment?
2. Generally well done, consider revising some statements which are awkwardly worded throughout the discussion.
