# Supplementary material for: Prevalence and socio-demographic correlates of accelerometer measured physical activity levels of school-going children in Kampala city, Uganda
Source: PLoS One. 2020 Jul 9;15(7):e0235211. doi: 10.1371/journal.pone.0235211 (PMC7347200; doi:10.1371/journal.pone.0235211)
Supplement: Attachment — Submitted filename: Rebuttal letter.docx [file pone.0235211.s004.docx]

**Rebuttal letter**

We are grateful to the editor and the two reviewers for their comments on our manuscript.

Please find below our responses to each point raised by the academic editor and reviewers. The manuscript has been edited, all typing and grammatical errors, and use of lower and upper case have been addressed. We have also included a paragraph on policy implications particularly joining and learning from global initiatives aimed at increasing children’s physical activity.

We believe that we have addressed all the concerns and our manuscript is ready for publication.

Sincerely,

On behalf of all the authors,

Bernadette Nakabazzi

***Academic Editor:***

No comments provided

**Reviewer #1**

A. ***Very well done with addressing my comments. There are some minor typos, grammatical errors, and issues with the use of uppercase and lowercase letters. I'm sure the editorial office will pick these issues up during copy editing and proofreading***.

We thank the reviewer for pointing out this, we have addressed the typing and grammatical errors, and the use of upper case and lower case throughout the manuscript.

B. ***I have (almost) no further comments; just a few suggestions for the authors to consider:***
1. ***If possible, avoid the use of "inactive" or "inactivity", instead, use "insufficiently active" or "insufficient activity". A healthy person cannot be inactive, I guess.***

We thank the reviewer for this suggestion, it has been corrected throughout the revised version. We have changed the words “inactive or Inactivity” to “insufficiently active or insufficient activity” and used them throughout the manuscript.

2. ***Please clarify the response rate issue - clearly state "The response rate was XX%". Consider adding a few sentences about a seemingly low-response rate in the discussion (limitations) section.***

We thank the reviewer for pointing this out we have clarified the response rate issue and clearly stated it as; “The response rate was 42.7% (line 199-200 in the revised version). Also the low response rate has been included in the study limitations (line 398 in the revised version).

3. ***In the Discussion section, please comment on the policy implications of these findings. How the results can be instrumental in informing active lifestyle strategies in Uganda, what policy initiatives may be required, how the government can align their efforts with the global community, e.g., WHO, Active Healthy Kids Global Alliance. The authors may wish to read Aubert et al. (2018) Global Matrix 3.0 Physical activity report card grades for children and youth: Results and analysis from 49 countries. Journal of Physical Activity and Health 15 (Supplement 2), S251-S273. I would encourage the authors to add a dedicated paragraph on "Implications for future research and policy" in the Discussion section before the Limitations of the study (no subheading required).***

   We thank the reviewer for suggesting this very important paper, we have included a paragraph in the discussion section on strategies to increase physical activity and policy implications of the current study results to read as;

“Therefore, there is need for developing effective strategies and policies with the aim of increasing physical activity levels among school going children in Kampala city and Uganda. This may be achieved by implementing strategies and policies that have been proposed by various global and regional organisations including those of the Active Healthy Kids Global Alliance (AHKGA) in the fight against the insufficient physical activity among children [81,82]. The current study further highlighted the need for nationally representative physical activity data. The Ministry of Education and Sports in Uganda should fund the development and release of a national report card on physical activity for children in Uganda for surveillance and promotion of physical activity among Ugandan children”.

**Reviewer # 2**

***The authors have sufficiently addressed all comments and questions I raised. I have no further questions nor concerns with this manuscript.***
